# Evaluation of sodium deoxycholate as solubilization buffer for oil palm proteomics analysis

**DOI:** 10.1371/journal.pone.0221052

**Published:** 2019-08-15

**Authors:** Benjamin Yii Chung Lau, Abrizah Othman

**Affiliations:** Malaysian Palm Oil Board, No 6, Persiaran Institusi, Bandar Baru Bangi, Kajang, Selangor, Malaysia; Universita degli Studi di Siena, ITALY

## Abstract

Protein solubility is a critical prerequisite to any proteomics analysis. Combination of urea/thiourea and 3-[(3-cholamidopropyl)dimethylammonio]-1-propanesulfonate (CHAPS) have been routinely used to enhance protein solubilization for oil palm proteomics studies in recent years. The goals of these proteomics analysis are essentially to complement the knowledge regarding the regulation networks and mechanisms of the oil palm fatty acid biosynthesis. Through omics integration, the information is able to build a regulatory model to support efforts in improving the economic value and sustainability of palm oil in the global oil and vegetable market. Our study evaluated the utilization of sodium deoxycholate as an alternative solubilization buffer/additive to urea/thiourea and CHAPS. Efficiency of urea/thiourea/CHAPS, urea/CHAPS, urea/sodium deoxycholate and sodium deoxycholate buffers in solubilizing the oil palm (*Elaeis guineensis* var. Tenera) mesocarp proteins were compared. Based on the protein yields and electrophoretic profile, combination of urea/thiourea/CHAPS were shown to remain a better solubilization buffer and additive, but the differences with sodium deoxycholate buffer was insignificant. A deeper mass spectrometric and statistical analyses on the identified proteins and peptides from all the evaluated solubilization buffers revealed that sodium deoxycholate had increased the number of identified proteins from oil palm mesocarps, enriched their gene ontologies and reduced the number of carbamylated lysine residues by more than 67.0%, compared to urea/thiourea/CHAPS buffer. Although only 62.0% of the total identified proteins were shared between the urea/thiourea/CHAPS and sodium deoxycholate buffers, the importance of the remaining 38.0% proteins depends on the applications. The only observed limitations to the application of sodium deoxycholate in protein solubilization were the interference with protein quantitation and but it could be easily rectified through a 4-fold dilution. All the proteomics data are available via ProteomeXchange with identifier PXD013255. In conclusion, sodium deoxycholate is applicable in the solubilization of proteins extracted from oil palm mesocarps with higher efficiency compared to urea/thiourea/CHAPS buffer. The sodium deoxycholate buffer is more favorable for proteomics analysis due to its proven advantages over urea/thiourea/CHAPS buffer.

## Introduction

Palm oil remains the most efficient oil crop in the world based on its land use (0.36% of the world agricultural land) and productivity (34% of world oils and fats production) [1]. There has been an increasing interest in studying the oil palm proteome to answer many physiological questions, for instance, the machinery of fatty acid production [2–6], fungal disease affecting the oil palm plantations [7, 8] and the flowering process [9] to enhance the sustainability of oil palm. Our proteomics studies revolved around establishing a quantitative model for oil palm lipid metabolism that would coincide with the biochemical, genomics and transcriptomics analyses. Previously, the oil palm transcriptomic studies have revealed elevated transcripts of several fatty acid biosynthetic enzymes in the fruit mesocarp, that lead to an increase in lipid production [10–15]. Expression of the proteins related to fatty acid production were also reported to be distinctive throughout oil palm development stages [2, 4]. Integration of these omics datasets could be exploited as a platform to further scrutinize the oil palm fruit mesocarp in order to comprehend the exact regulation control of high-value fatty acid production in the effort to optimize the economic value and sustainability of palm oil.

Proteomics techniques have been routinely utilized to study protein compositions and cellular functions of plants, animals and microorganisms [16]. One of the prerequisites to an effective proteomics analysis is a good protein solubility [17]. However, it is well known that extracts from plant materials, such as oil palm origin, consists of contaminants like phenolics, polyphenols and lipids. These contaminants strongly interfere with subsequent protein extraction process [12, 18–20]. One of the typical interferences is the inability for the proteins to dissolve completely after protein enrichment with trichloroacetic acid/acetone or ammonium acetate/methanol [21–26]. A complete dissolution of proteins in any given sample is highly crucial to enable further downstream mass spectrometric analyses. The use of different buffers, detergents and surfactants to dissolve proteins depend strictly on their compatibility with downstream proteomics analyses. Many studies have employed denaturing buffers containing guanidine hydrochloride [27, 28], urea and/or thiourea to solubilize proteins from recalcitrant tissues [29–31]. Although sodium dodecyl sulfate has the strongest solubilization power, this detergent is incompatible with protease activity and mass spectrometry [32, 33]. Meanwhile, some of the major drawbacks of urea/thiourea are the resulting additional carbamylation modification of *N*-termini and lysine residues, resulted in raising the false discovery rate of identified proteins [34–37] and its incompatibility with tryptic digestion at high concentration [38]. Guanidine hydrochloride could be used with endoprotease Lys-C but not with a more routine trypsin for protein digestion due to inhibition of the digestion enzyme [36, 39]. Another less common detergents like sodium deoxycholate is widely used to solubilize membrane proteins [40–43], in addition to improving protein digestions in some studies due to its compatibility with mass spectrometry [44–48]. However, there has been no documented work until now that described the use of sodium deoxycholate in solubilizing proteins extracted from recalcitrant and oily plant tissues such as oil palm fruit mesocarps.

This study systemically evaluated the solubilization property of sodium deoxycholate as a potential alternative to other solubilization agents such as urea, thiourea and 3-[(3-cholamidopropyl)dimethylammonio]-1-propanesulfonate (CHAPS) for proteins extracted from the fruit mesocarp of *Elaeis guineensis*. The evaluation was carried out by comparing the total protein yields, one-dimensional gel electrophoresis profile and shotgun proteomics analysis as the metric of validation.

## Materials and methods

### Plant materials

Oil palm fruit bunches (of 20^th^ week after anthesis) from the standard Dura x Pisifera oil palm crosses (*Elaeis guineensis* var. Tenera progeny) were used in this study. The mesocarps from the randomly selected fruitlets from each bunch were sliced, snap frozen in liquid nitrogen and stored at– 80°C.

### Protein extraction

Proteins were extracted according to Lau and co-workers with some modifications [49]. 10 g of sliced mesocarps were ground and mixed well with 25 mL of cold acetone containing 10% trichloroacetic acid and 1 mM dithiothreitol on ice. The slurry was then centrifuged at 13,000 g for 10 min at 4°C (RA-300 rotor, Kubota 7820, Kubota Corporation, Tokyo, Japan). The washing step was repeated once before adding 25 mL of cold 80% methanol containing 0.1 M ammonium acetate to the precipitate; mixed well and centrifuged as before, on ice. The precipitated mesocarp pellet was washed with 25 mL of cold 80% acetone. The mixture was mixed well and centrifuged again at 13,000 g for 10 min at 4°C. Pellet was gently re-suspended in 15 mL of extraction buffer containing 0.7 M sucrose, 1 M Tris-HCl, pH 8.3, 5 M NaCl, 50 mM DTT, 1 mM EDTA and a tablet of Roche protease inhibitors. The resuspension was sonicated using ultrasonic bath for 30 mins (Townson & Mercer Ltd., England, UK). The mixture was then sieved through two layers of Miracloth (Calbiochem, EMB Millipore Corporation, Billerica, MA) to separate non-macerated plant materials. An equal volume of fresh 50 mM, pH 8.0 Tris-saturated phenol (15 mL) was added to the mixture, mixed well and centrifuged at 15,000 g for 15 min at 4°C (RA-300 rotor, Kubota 7820) for phase separation. Proteins in the upper phase were precipitated by adding five volumes of cold ammonium acetate-saturated methanol (25 mL) to one volume of phenol phase, mixed well and incubated at -20°C overnight before being centrifuged at 15,000 g for 15 min at 4°C (RA-300 rotor, Kubota 7820). The protein pellets were then rinsed with 5 mL of cold ammonium acetate-saturated methanol and washed three times with 5 mL of cold 80% acetone. The protein pellet was air-dried for 5 min.

#### Solubilization of protein pellet

In this study, four different solubilization buffers were used to solubilize the ammonium acetate/methanol precipitated proteins. A volume of 600 μL of each evaluated buffers was added to the protein pellet. *Buffer A*: *Urea/thiourea/CHAPS*– 7 M urea, 2 M thiourea, 4% CHAPS, 0.4% DTT, 10 mM Tris base; *Buffer B*: *Urea/CHAPS*—7 M urea, 4% CHAPS, 0.4% DTT, 10 mM Tris base; *Buffer C*: *Urea/sodium deoxycholate*– 7 M urea, 4% sodium deoxycholate, 0.4% DTT, 10 mM Tris base; *Buffer D*: *Sodium deoxycholate*– 4% sodium deoxycholate, 0.4% DTT, 10 mM Tris base. Commercially available 2D Quant Kit (GE Healthcare Life Sciences, Uppsala, Sweden) was then utilized to determine protein content in the samples. Bovine serum albumin provided with the kit was used as the protein calibration standard and each quantitation was performed in duplicate. A 4-fold dilution was performed on the proteins solubilized with sodium deoxycholate-containing buffers when Pierce 660 nm Protein Assay Reagent (Thermo Scientific, IL, USA) or Coomassie-based Bradford was used. Without the dilution, precipitated sodium deoxycholate would interfere with the absorbance readings.

#### In-solution protein digestion

Protein digestion was performed according to Lau and co-workers [3, 4]. To obtain 50 μg of proteins for digestion, solubilized proteins in Buffer A, B and C were re-precipitated with cold ammonium acetate-saturated methanol. The precipitated proteins were re-suspended in 0.1 M ammonium bicarbonate and 1 M urea before reduction and alkylation using 50 mM tris(2-carboxyethyl)phosphine and 150 mM iodoacetamide, respectively. Sodium deoxycholate (1% w/v) was added to the protein solution prior to digestion with 4 μg of modified sequencing grade trypsin (Promega, Madison, WI, USA) in 50 mM NH_4_HCO_3_ for 16 h at 37°C. Sodium deoxycholate was removed after tryptic digestion by acidification using 0.5% formic acid and centrifugation at 14 000 g (RA-300, Kubota 7820) for 15 min at ambient temperature. The peptide solution was then dried in a centrifugal evaporator (CentriVap Concentrator, Labconco, MO, USA). *Peptide clean-up–*The dried peptide pellet was resuspended in 200 μL of 0.1% formic acid. Acetonitrile, methanol and 0.1% formic acid-conditioned Empore solid phase extraction disks (3M Purification, Inc., MN, USA) were added to the peptide solution and incubated at ambient temperature with slight agitation for 4 h. The bound peptides on the C18 membrane disks were sequentially eluted with 50% ACN in 0.1% FA for 2.5 h.

#### One-dimensional gel electrophoresis

To obtain 100 μg of proteins for electrophoretic separation, the solubilized proteins were re-precipitated with cold ammonium acetate-saturated methanol. The precipitated proteins were then dissolved in Laemlli buffer (62.5 mM Tris-HCl, pH 6.8, 2% SDS, 25% glycerol, 0.01% bromophenol blue, 0.005% β-mercaptoethanol) and denatured by boiling at 95°C for 4 min [3]. 100 μg protein was loaded into each lane on a 1.0 mm in-house casted 12% polyacrylamide gel. Electrophoresis was conducted in a Bio-Rad mini-PROTEAN Tetra Cell apparatus (Bio-Rad Laboratories Inc., Hercules, CA) at 200 V for 1 h. Following electrophoresis, the separated proteins were fixed for 30 min in a fixing solution (50% ethanol, 10% acetic acid) and stained with an in-house prepared Colloidal Coomassie G-250. The gel was destained with Milli-Q water until the gel background was clear. The gel was scanned as digital image using Bio-5000 Plus scanner (Microtek, Hsinchu, Taiwan) according to the manufacturer’s instructions.

#### Liquid chromatography-tandem mass spectrometry

Separation and spectra acquisition of the protein digests was conducted with an EASY-nano liquid chromatography (EASY-nLC) 1200 System (Thermo Scientific, MA, USA), coupled to a Q Exactive Plus Hybrid Quadrupole-Orbitrap mass spectrometer (Thermo Scientific, MA, USA). Tryptic digests were reconstituted in 20 μL of 0.1% FA and 5% ACN. A sample volume of 2 μL was injected into an Acclaim PepMap 100 C18 reversed phase column (3 μm, 0.075 x 150 mm) (Thermo Scientific, MA, USA) for peptide separation. The column was equilibrated with 95% mobile phase A (0.1% FA) and 5% mobile phase B (0.1% FA in ACN). A gradient of 5–35% mobile phase B in 70 min was employed to elute the bound peptides at a flow rate of 300 nL min^-1^. Gas-phase peptide ions were generated by electrospray ionization using a spray voltage of 1800 V. Peptide precursors survey scan was acquired in the Orbitrap mass analyzer with a mass range of *m/z* 310–1800 and resolving power of 70,000. Maximum injection time applied was 100 ms. Peptide precursors with charge state of 2–8 were chosen for tandem MS (MS^2^). Tandem MS conditions consisted of rapid scan rate with the linear ion trap mass analyzer using a resolving power of 17,500, 0.7 *m/z* isolation window and an maximum injection time of 60 ms. Precursors were fragmented using collision-induced and high-energy collision-induced (CID and HCD) at a normalized collision energy of 28%, respectively. Mass range scanned was from *m/z* 110–1800. The mass spectrometry proteomics data have been deposited to the ProteomeXchange Consortium via the PRIDE [50] partner repository with the dataset identifier PXD013255 and 10.6019/PXD013255.

#### Data analysis

Data acquisitions in positive mode were executed with Thermo Scientific Xcalibur (Version 4.1.31.9) (Thermo Scientific, MA, USA). Generated raw data (.RAW) was processed with Thermo Scientific Proteome Discover, version 2.1 (Thermo Scientific, MA, USA) to generate peak lists in .DTA format for database searching. Tandem (MS^2^) mass spectra were searched with SEQUEST HT engine against *Elaeis guineensis* (TaxID = 51953) and *Phoenix dactylifera* (TaxID = 42345) taxonomies (containing 35,972 and 33,101 protein sequences, respectively, as of 30^th^ October 2017) in NCBI protein database. Mass tolerances for peptide and product ions were set to 20 ppm and 0.5 Da. Trypsin was designated as the protease with two missing cleavages allowed. Carbamidomethylation on cysteine and lysine was set as the fixed modification while oxidation of methionine and deamidation of asparagine and glutamine were searched as variable modifications. Proteins were accepted if they had at least one Rank 1 peptide. A decoy database contained randomized sequences of searched taxonomies. All database searches were also performed against the decoy database to determine the false discovery rate. All peptide spectral matches were validated using the Percolator version 2.04 (component of Proteome Discover) based on *q*-value at a 1% false discovery rate. Venn diagram of the identified proteins from the evaluated solubilization buffers was created using a free web-based program (http://bioinformatics.psb.ugent.be/webtools/Venn/). Biological process, cellular component and molecular function of the identified proteins were annotated using the Retrieve/ID mapping tool in Uniprot (https://www.uniprot.org/uploadlists/). Gene ontology (GO) terms associated with the identified proteins from all the evaluated solubilization buffers were collected from the Uniprot-GOA database (http://www.ebi.ac.uk/GOA).

#### Supervised partial least squares-discriminant analysis (PLS-DA)

Supervised PLS-DA using MetaboAnalyst 4.0 (http://www.metaboanalyst.ca/) [51] was employed to determine the correlation of the identified proteins (based on their peak intensities) and different solubilization buffers. Data inputs containing measured *m/z* value for each peptide and their corresponding retention time and intensities were extracted from the Thermo RAW files. Four replicates representing each of the evaluated solubilization buffers were used (total of 93,777 peaks, with an average of 5861.1 peaks per sample). Peaks of the same group were summed, if they are from one sample, resulting in 5,483 peak groups. For peak matching, these variables were grouped based on their retention time. Mass and retention time were set at 0.025 *m/z* and 30 secs, respectively. Interquartile range (IQR) filtered out the unusable variables [52] to improve the regression model. These variables are normally the uninformative regions or noise of mass spectra. Normalization and data scaling based on data dispersion were performed using the sum of intensities and Pareto scaling [53]. Normalization of the datasets improves the interpretability of the model. Pareto scaling (square root of the standard deviation as the scaling factor) was applied because of the dynamism of the proteomics datasets [54, 55]. Statistical model was validated using permutation test as PLS-DA tends to over fit data [56, 57]. This test determined if the differences between the evaluated buffers were significant. In the permutation test, the *Y*-block (class assignment) was permutated 1000 times. For every PLS-DA model built, a sum of squares between/within (B/W) ratio was calculated for the class assignment predictions. These ratios were plotted in a histogram. The further to the right the B/W ratio of the original class assignment to the distribution based on the permuted class assignment, the more significant the contrast between the two class assignments from a statistical point of view.

All laboratory protocols had been deposited in protocols.io. http://dx.doi.org/10.17504/protocols.io.434gyqw [PROTOCOL DOI].

## Results and discussion

Proteomics studies are critically dependent on soluble and good quality proteins. The study was conducted to determine the efficiency of sodium deoxycholate (SDC) in solubilizing oil palm mesocarp proteins compared to other urea/CHAPS-containing buffers. The efficiency was compared in terms of their total protein yields, electrophoretic patterns, chromatographic and mass spectra patterns, as well as number of identified proteins and their resulting gene ontologies. A statistical analysis using partial least squares-discriminant analysis (PLS-DA) was also incorporated into the evaluation criteria to determine the variability of the solubilization buffers. In this study, comparisons were made between SDC and a routinely used urea/thiourea/3-[(3-cholamidopropyl)dimethylammonio]-1-propanesulfonate (CHAPS) buffers for solubilization of proteins derived from oil-rich plant tissues such as oil palm [29–31]. Effect of SDC in replacing CHAPS (in urea/SDC and urea/CHAPS buffers) were also compared to evaluate the different detergents and the complimentary of urea/SDC in protein solubilization efficiency.

The first criteria to determine the efficiency of SDC as solubilization buffer was to investigate the total protein yields after solubilization in different buffers. The protein quantitation for all the proteins solubilized in urea/thiourea/CHAPS, urea/CHAPS, urea/SDC and SDC buffers was repeated three times. As shown in S1 Fig, the solubilization power of all buffers tested was quite satisfactory and no extensive loss in protein yield was recorded. Protein yield from urea/thiourea/CHAPS buffer was 1.13 ± 0.07 μg/μL. Protein yield from urea/CHAPS buffer was 1.17 ± 0.11 μg/μL. Meanwhile, protein yields from urea/SDC and SDC buffers decreased to 0.90 ± 0.07 μg/μL and 0.86 ± 0.05 μg/μL, respectively (compared to urea/thiourea/CHAPS). The total protein yield for SDC buffer was 0.86 ± 0.05 μg/μL. This was a 0.27 μg/μL reduction compared to the urea/thiourea/CHAPS buffer. Clearly, the presence of strong chaotropic agents and detergent is unparalleled in their solubilization efficiency. Meanwhile, the combination of urea/SDC did not improve the solubilization efficiency as compared to urea/thiourea/CHAPS, urea/CHAPS or even SDC buffers. We would expect a contrasting effect of the combination as both urea and SDC are also chaotropic agent and surfactant. Thus, the results suggested that CHAPS was not substitutable by SDC as detergent. Our observations also established that prior to protein quantitation, the assays containing SDC surfactant needed to be diluted about 4-fold (< 1% SDC) to avoid interference to the absorbance reading (data not shown). Unless the proteins were precipitated before the quantitation assay, or GE 2-D Quant kit was used to determine the protein content, this step is critical to achieve accurate and reproducible protein yield.

Qualitative comparison of the solubilization efficiencies using polyacrylamide gel showed that proteins solubilized by all the buffers were separated into well resolved and good intensity bands without any apparent sign of degradation or interference due to impurities (S2 Fig). Relative number of protein bands was identical except for proteins solubilized in urea/SDC buffer. The important pattern shown by these data was that, although solubilized proteins in SDC buffer resulted in lower yield compared to urea/thiourea/CHAPS buffer, majority of the solubilized proteins from both buffers were still detected in gel. However, a number of bands were missing for SDC buffer, as indicated in S2 Fig. That might explain the lower total protein yield for SDC buffer compared to urea/thiourea/CHAPS and urea/CHAPS buffers. Meanwhile, electrophoretic profile of urea/CHAPS and urea/SDC buffers showed a reduction in the number of protein band for the latter. The combination effect of urea and SDC seemed to lower the number of separated proteins on polyacrylamide gel or reduce the band intensities. This electrophoretic pattern profile was in agreement with the protein yield measurements obtained earlier for both solubilization buffers. We deduced that the possible reason might be due to interference from an incomplete removal of high concentration of SDC (4%) prior to gel electrophoresis but more works to elucidate this observation was necessary.

**Fig 1:**
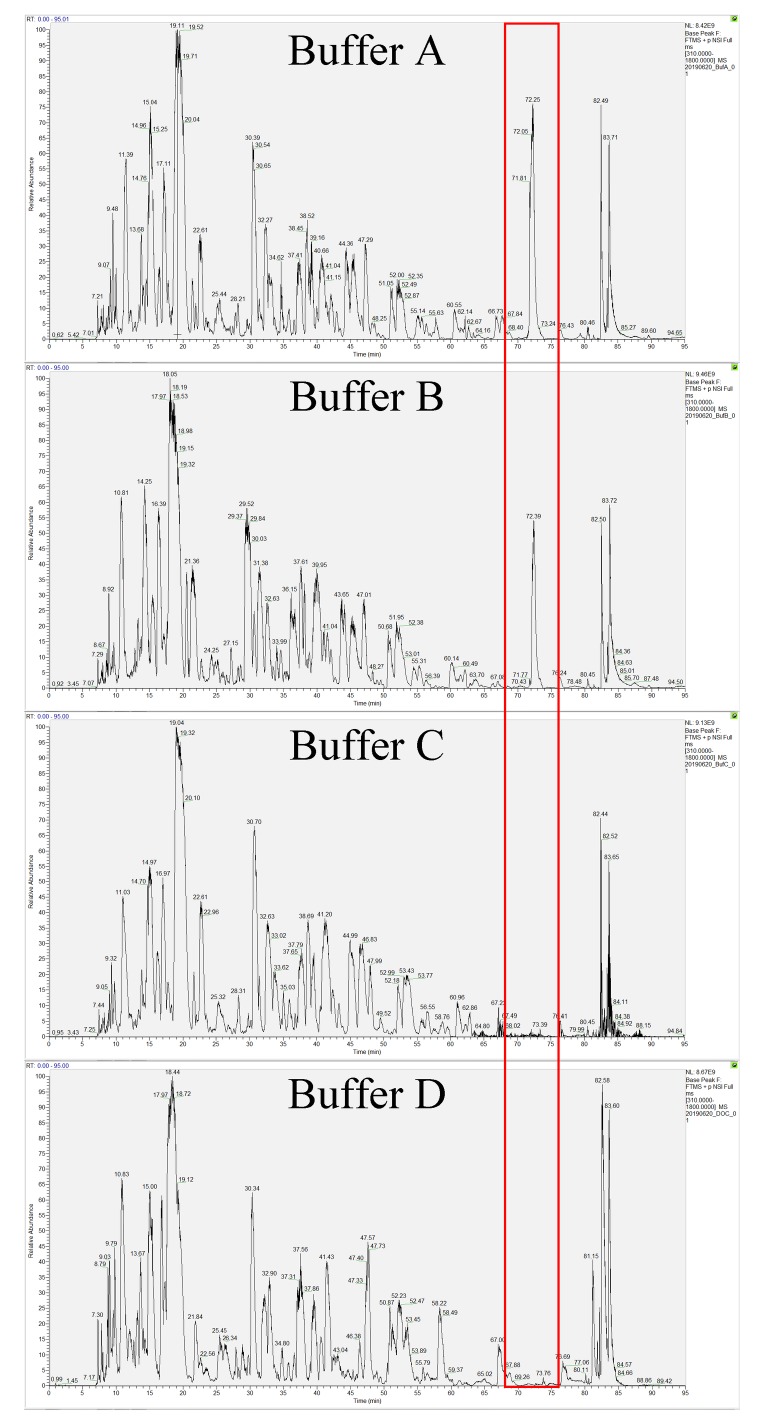
Base peak chromatogram of peptides digested from the four solubilization buffers (A-D, from top). Red box indicate the contaminant peaks (CHAPS). Buffer A: Urea/thiourea/CHAPS; Buffer B: Urea/CHAPS; Buffer C: Urea/Sodium deoxycholate; Buffer D: Sodium deoxycholate.

The solubilized proteins were subsequently tryptic digested and analyzed mass spectrometrically. An EASY-nano liquid chromatography (EASY-nLC) 1200 System (Thermo Scientific, MA, USA), coupled to a Q Exactive Plus Hybrid Quadrupole-Orbitrap mass spectrometer (Thermo Scientific, MA, USA) was used to detect the separated peptides. Base peak chromatograms for the separated peptides from the four different solubilization buffers were presented in Fig 1. Comparison of the chromatograms for all the tested solubilization buffers revealed similar profiles. However, unlike the urea/SDC and SDC buffers, proteins solubilized in urea/thiourea/CHAPS and urea/CHAPS buffers gave a signature peak at approximately 72 minutes into the chromatographic separation (indicated with a red box in Fig 1). Complete removal of excess CHAPS was challenging although ammonium acetate-saturated methanol had removed most of the CHAPS (performed prior to protein digestion). As a result, an intense peak ion would still be noticeable at 615 *m/z* (MH^+^) in the mass spectra (fragmentation of peak at 72 minutes). However, this particular contaminant peak was not detected with urea/SDC and SDC buffers given that CHAPS was not added. Although CHAPS had been reported to prevent protein loss through precipitation or aggregation [58], this was not observed in this study as major peaks were present in all of the evaluated buffer chromatograms. The relative abundance of the ions was also comparable among all the solubilization buffers.

**Fig 2:**
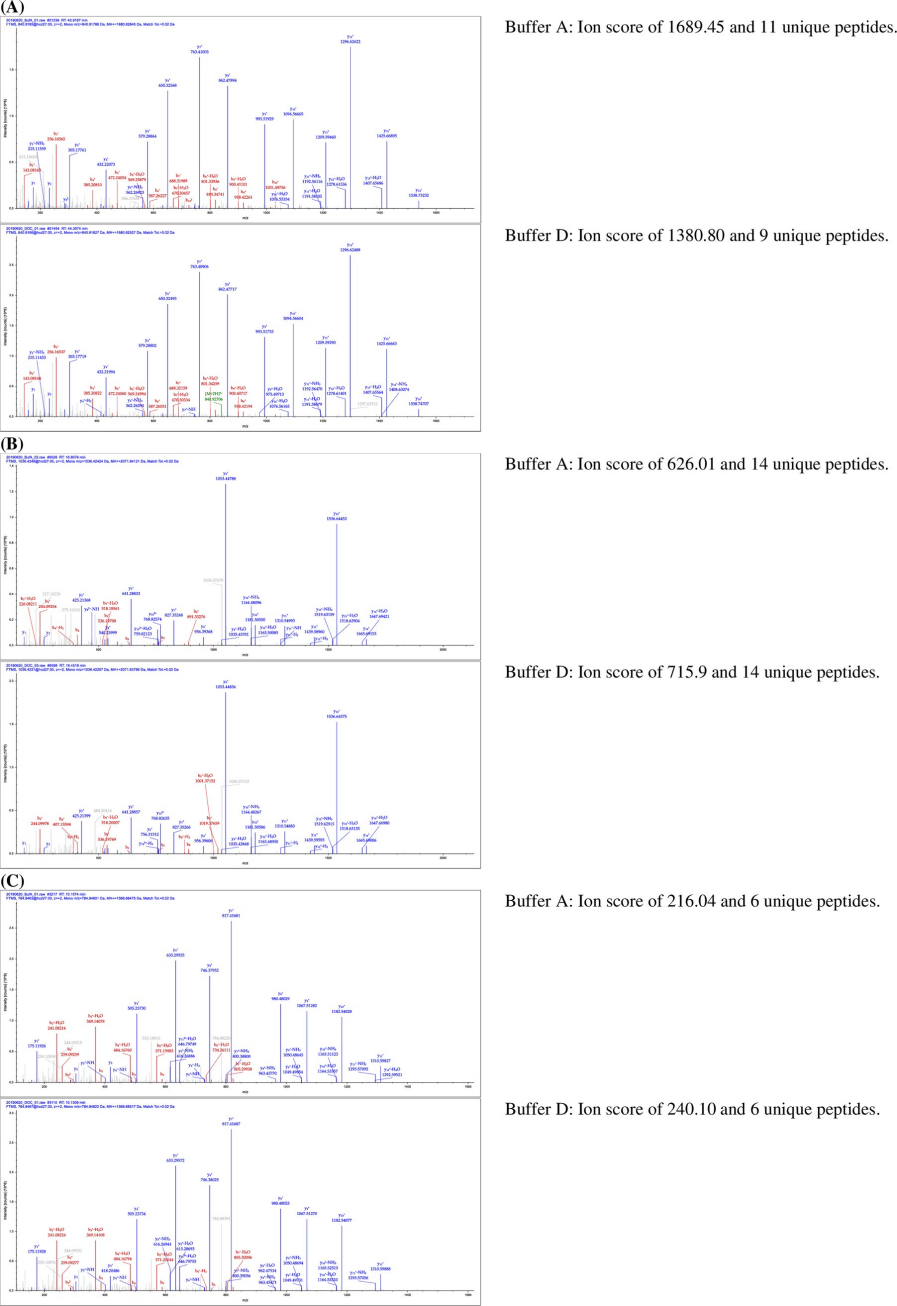
Tandem (MS/MS) mass spectra of peptides showing the b and y ions series. (A) Peptide AALESDTMVLAFEAGR corresponds to protein enoyl-ACP reductase; (B) Peptide KGGEYEPEEQPEADTDYSR correponds to protein phospholipase D alpha 1; (C) Peptide EEQDSYAIQSNER corresponds to protein acetyl-CoA acetyltransferase.

A more in-depth comparison between urea/thiourea/CHAPS and SDC buffers was made using three representative peptides corresponded to some of the targeted fatty acid biosynthetic enzymes in the oil palm proteomics works. For this study, the peak intensities, ion scores, detected unique peptides and the coverage of *b* and *y* ion series of these representative peptides were compared (Fig 2). Peptide AALESDTMVLAFEAGR, with a SEQUEST ion score of 1689.45 was identified to enoyl-ACP reductase in urea/thiourea/CHAPS buffer (Fig 2A). In total, 11 unique peptides were identified to enoyl-ACP reductase. With SDC buffer, slightly lower SEQUEST ion score (1380.80) and total number of unique peptides (9) were acquired. More importantly, the coverage of *b* and *y* ions for AALESDTMVLAFEAGR from both buffers was similar. Nevertheless, the *b* and *y* ion intensities for AALESDTMVLAFEAGR in SDC buffer were relatively higher. An ideal MS/MS spectrum would have high signal to noise ratio and contain all the *N*-terminal *b* ions and *C*-terminal *y* ion fragments, as observed with the described peptides detected in urea/thiourea/CHAPS and SDC buffers. Fig 2 also showed the comparison of another two peptides detected from urea/thiourea/CHAPS and SDC buffers in term of their SEQUEST ion scores, unique peptides and coverage of *b* and *y* ions. Both peptide KGGEYEPEEQPEADTDYSR and EEQDSYAIQSNER corresponded to phospholipase D alpha 1 and acetyl-CoA acetyltransferase, respectively. Ion score for peptide KGGEYEPEEQPEADTDYSR was increased in SDC buffer (715.90) relatively to urea/thiourea/CHAPS buffer (629.01) (Fig 2B). Total detected unique peptides for phospholipase D alpha 1 in both buffers remained at 14 peptides. In another comparison, ion score for peptide EEQDSYAIQSNER for both buffers were almost similar at 216.04 (urea/thiourea/CHAPS) and 240.10 (SDC buffer). The total number of detected unique peptides was the same at 6 for both buffers (Fig 2C). In both cases, their *b* and *y* ion coverages remained similar but with relatively higher intensities for SDC buffer. Acetyl-CoA acetyltransferase, enoyl-ACP reductase and phospholipase D are important enzymes for initiation, synthesis of fatty acids and their metabolism, respectively [59]. They are of interest in the proteomics studies of oil palm mesocarps and therefore, it is crucial to be able to identify them using high quality mass spectra regardless of the solubilization buffers used for oil palm mesocarp proteins.

**Fig 3:**
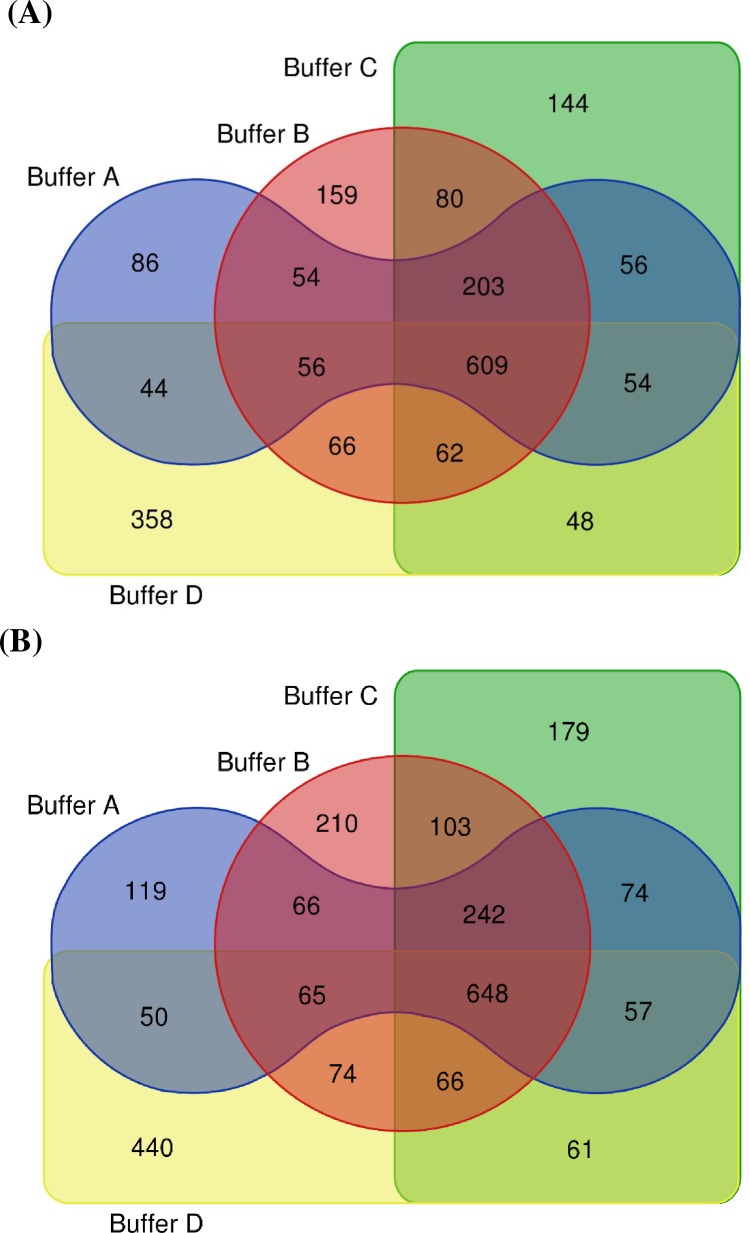
Venn diagrams of (A) proteins and (B) peptides, searched and identified using SEQUEST-HT protein search engine from four different solubilization buffers (Buffer A-D). Buffer A: Urea/thiourea/CHAPS; Buffer B: Urea/CHAPS; Buffer C: Urea/Sodium deoxycholate; Buffer D: Sodium deoxycholate.

Proteomics analysis was subsequently performed on the oil palm mesocarp proteins solubilized in four different solubilization buffers. A total of 1161 proteins (3040 peptides) was identified from urea/thiourea/CHAPS buffer compared to 1296 proteins (3461 peptides) from SDC buffer. Urea-induced carbamylation on lysine residues was found on 225 peptides from urea/thiourea/CHAPS buffer. In contrast, only 44 peptides were modified on the same amino acid in SDC buffer, a reduction of 67.3%. Removal of thiourea did not affect the protein solubilization significantly as 1288 proteins (3569 peptides) were identified from urea/CHAPS buffer. The combination of urea/SDC appeared to reduce the number of identified proteins by 1.29% only (1255 proteins, 3382 peptides), compared to urea/CHAPS buffer. Of the total identified peptides, carbamylation on lysine residue occurred on 356 and 336 peptides for urea/CHAPS and urea/SDC buffers, respectively. The outcome of the proteomics analysis clearly strengthened the results acquired from their protein quantitation assays (S1 Fig) and one-dimensional gel electrophoresis profile (S2 Fig). Furthermore, the modification search revealed that peptide carbamylation had occurred in all buffers involving urea, with varying degrees. Results from further examination of the protein (Fig 3A) and peptide (Fig 3B) identifications were shown in four-way Venn diagrams. Urea/thiourea/CHAPS and SDC buffers both shared 763 proteins in common (62.0% of total identified proteins). About 34.0–41.2% of the total identified proteins from urea/thiourea/CHAPS (399) and SDC (534) buffers were unique to each buffer. Urea/thiourea/CHAPS and SDC buffers both shared 820 peptides while 501 and 641 peptides were unique to each respective buffer. 954 or 75.0% of the total identified proteins from urea/CHAPS and urea/SDC buffers were shared. Urea/CHAPS and urea/SDC buffers had 335 (415 unique peptides) and 302 unique proteins (371 unique peptides), respectively.

Urea/thiourea/CHAPS had better solubilization efficiency than SDC buffer. However, the proteomics results indicated that SDC buffer was able to elevate the total identified proteins to a greater extent, although only 62.0% of the total identified proteins were shared between the buffers. Depending on the biological questions to be elucidated, the remaining 38.0% identified proteins might not be crucial, at least not in the oil palm proteomics studies. Further works are in progress to look into these unique proteins [60]. The differences could be due to the characteristic of urea/thiourea/CHAPS in disrupting hydrogen bonds and hydrophobic interactions of the proteins for solubilization. As mentioned by Broeckx and co-workers [61], protein crosslinking reversion was improved in an alkaline environment. Therefore, a slightly basic environment provided by a fresh urea/thiourea/CHAPS buffer might facilitate the protein solubilization. Unlike urea/thiourea/CHAPS, SDC is a deoxycholic acid derivative. However, the basic environment in the SDC buffer was conferred by the addition of Tris. The presence of dithiothreitol could also assist in the reduction of internal disulfide bonds. In the assessment of SDC as a detergent substitute, SDC was evidently not able to perform as effectively as CHAPS (in urea buffer) based on the number of identified proteins. Unlike SDC, CHAPS could protect the protein activity due to its zwitterionic characteristic while SDC might induce denaturation of the proteins to some extent [62–64]. This was a very likely reason as to the slight differences observed in this study relating to the number of identified proteins and peptides.

**Fig 4:**
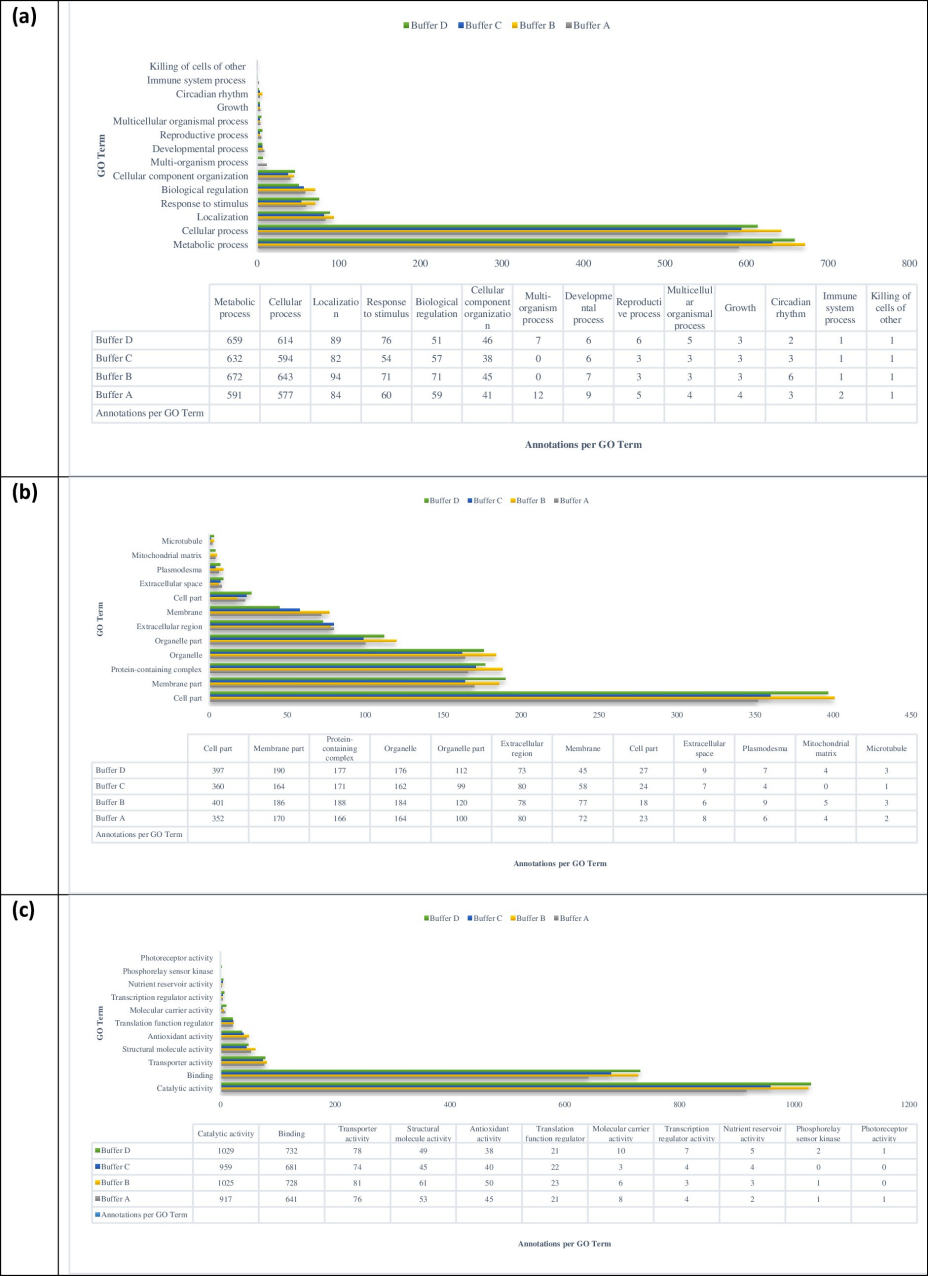
Biological process of proteins solubilized using four different solubilization buffers (A-D). GO terms associated with the identified proteins were collected from the Uniprot-GOA database (http://www.ebi.ac.uk/GOA). Buffer A: Urea/thiourea/CHAPS; Buffer B: Urea/CHAPS; Buffer C: Urea/Sodium deoxycholate; Buffer D: Sodium deoxycholate.

To further evaluate the efficiency of the solubilization buffers, identified proteins from all the buffers were categorized according to their biological processes, subcellular localizations and molecular functions (Fig 4). All proteins identified were annotated with same gene ontology terms regardless of the solubilization buffers used. Majority of the gene ontology terms for both SDC and urea/CHAPS buffers were higher relatively, compared to urea/thiourea/CHAPS and urea/SDC buffers (Fig 4A). In most biological processes, number of annotated proteins from urea/CHAPS buffer was slightly higher compared to SDC buffer (except in response to stimulus, cellular component organization, multiorganism processes and reproductive process). Note that number of identified proteins for both buffers were comparable (1296 proteins for SDC and 1288 for urea/CHAPS, respectively) and higher relatively to the rest of the evaluated buffers. Majority of the proteins were involved in metabolic and cellular processes. Fig 4B illustrates the cellular components of the identified proteins. In overall, proteins from all the buffers were annotated with the same cellular localization. Most proteins were located in cell, followed by membrane, protein-containing complex and organelles. Least proteins were localized in the extracellular, plasmodesma, mitochondrial matrix and microtubule. Molecular activity of the proteins identified was also classified using gene ontology analysis (Fig 4C). There were 11 activities associated with all the proteins from the different solubilization. Most proteins were implicated in binding and catalytic activities. Less than 10 proteins were associated with transcription regulator, nutrient reservoir, phosphorelay sensor kinase and photoreceptor activities.

**Fig 5:**
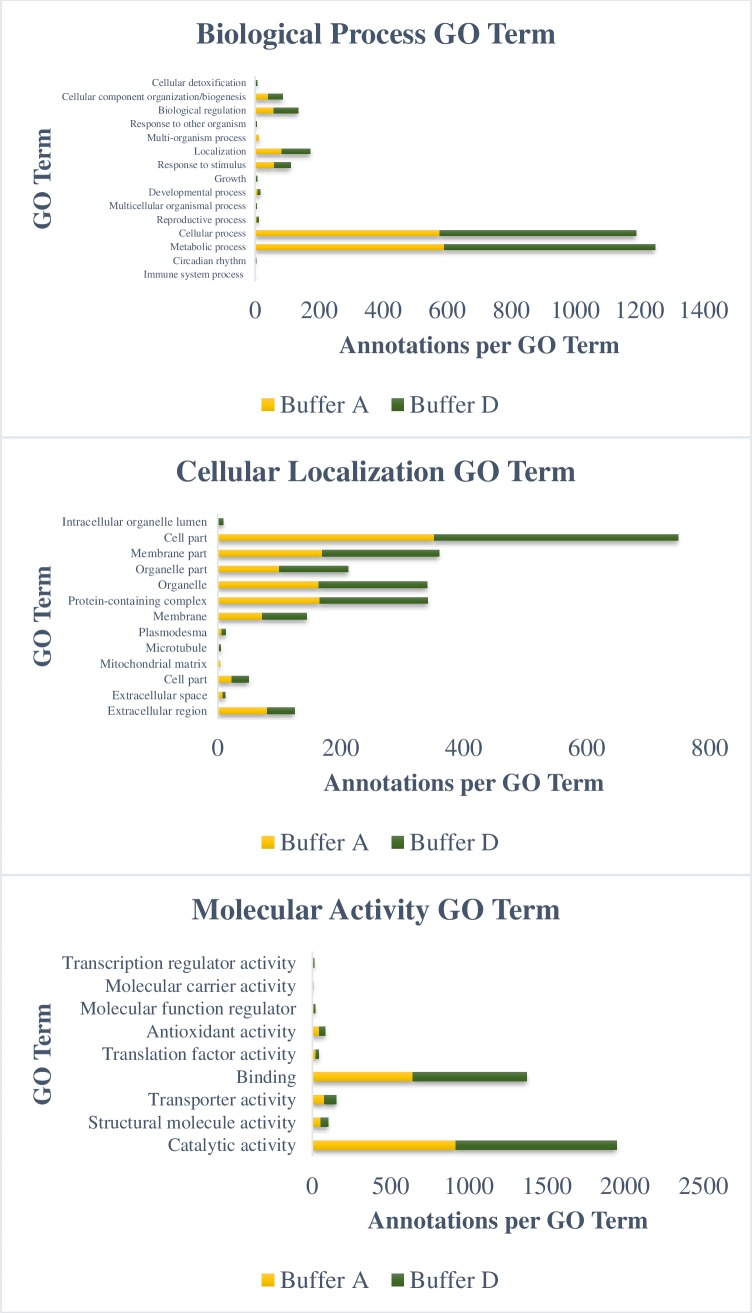
Comparison of biological process, cellular localization and molecular activity of proteins solubilized in Buffer A (urea/thiourea) and Buffer D (sodium deoxycholate).

A more detailed comparison of the gene ontology for biological process, subcellular location and molecular activity was made on the identified proteins from urea/thiourea/CHAPS and SDC buffers (Fig 5). The comparisons revealed that SDC buffer had profound effects on the resulting proteome. In particular, SDC buffer had enriched proteins in every functional categories. The enrichment in the biological regulation and metabolic processes of protein identified using the SDC buffer, could contribute significantly to our efforts in understanding the regulation of oil palm fatty acid biosynthesis mechanism. In terms of cellular components, there was no obvious significant difference or additional gene ontology terms observed in the comparative analysis. Proteins localized in the membrane (5.6%) and cell (6%) were slightly enriched, which coincide with the use of SDC buffer. Molecular activities of the identified proteins from SDC buffer were also enriched, particularly catalytic activity (5.8%) and binding (6.6%).

**Fig 6:**
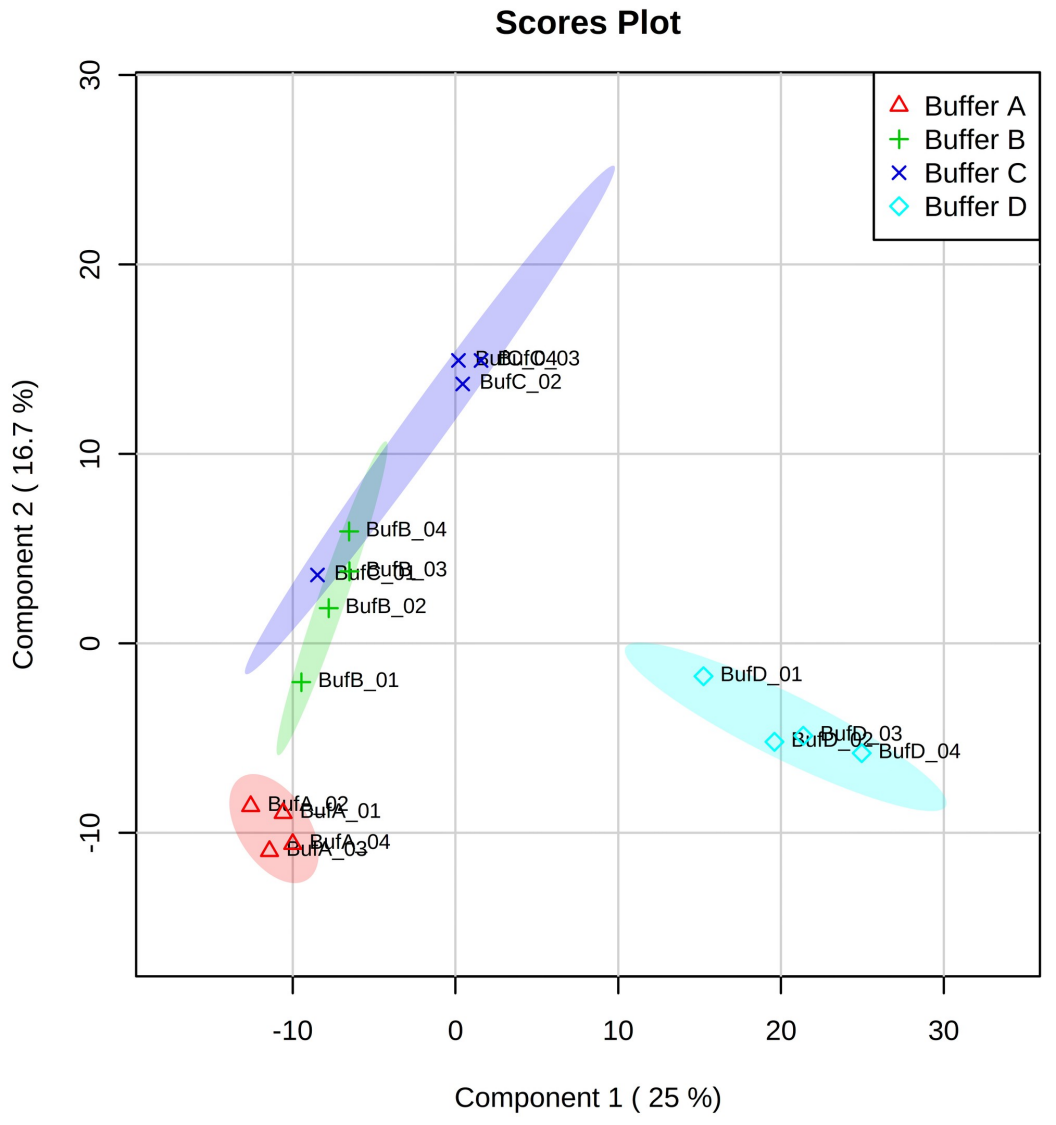
Scores plot from PLS-DA representing proteomics data from the evaluation of four different solubilization buffers. The plot displays the grouping of buffers used in this study (A-D). Buffer A: Urea/thiourea/CHAPS; Buffer B: Urea/CHAPS; Buffer C: Urea/Sodium deoxycholate; Buffer D: Sodium deoxycholate.

**Fig 7:**
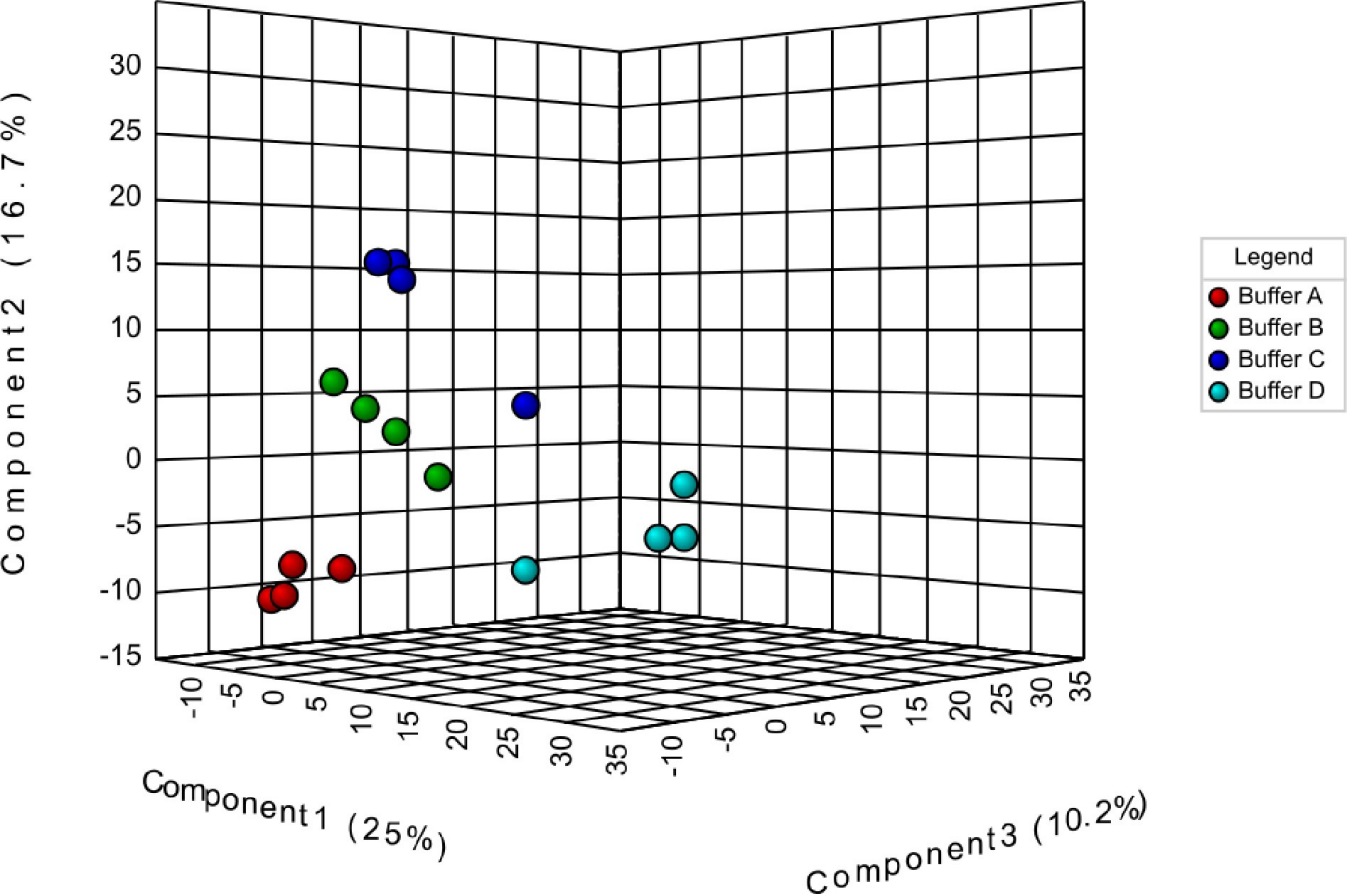
Three-dimensional scores plot from PLS-DA representing proteomics data from the evaluation of four different solubilization buffers. Buffer A: Urea/thiourea/CHAPS; Buffer B: Urea/CHAPS; Buffer C: Urea/Sodium deoxycholate; Buffer D: Sodium deoxycholate.

**Fig 8:**
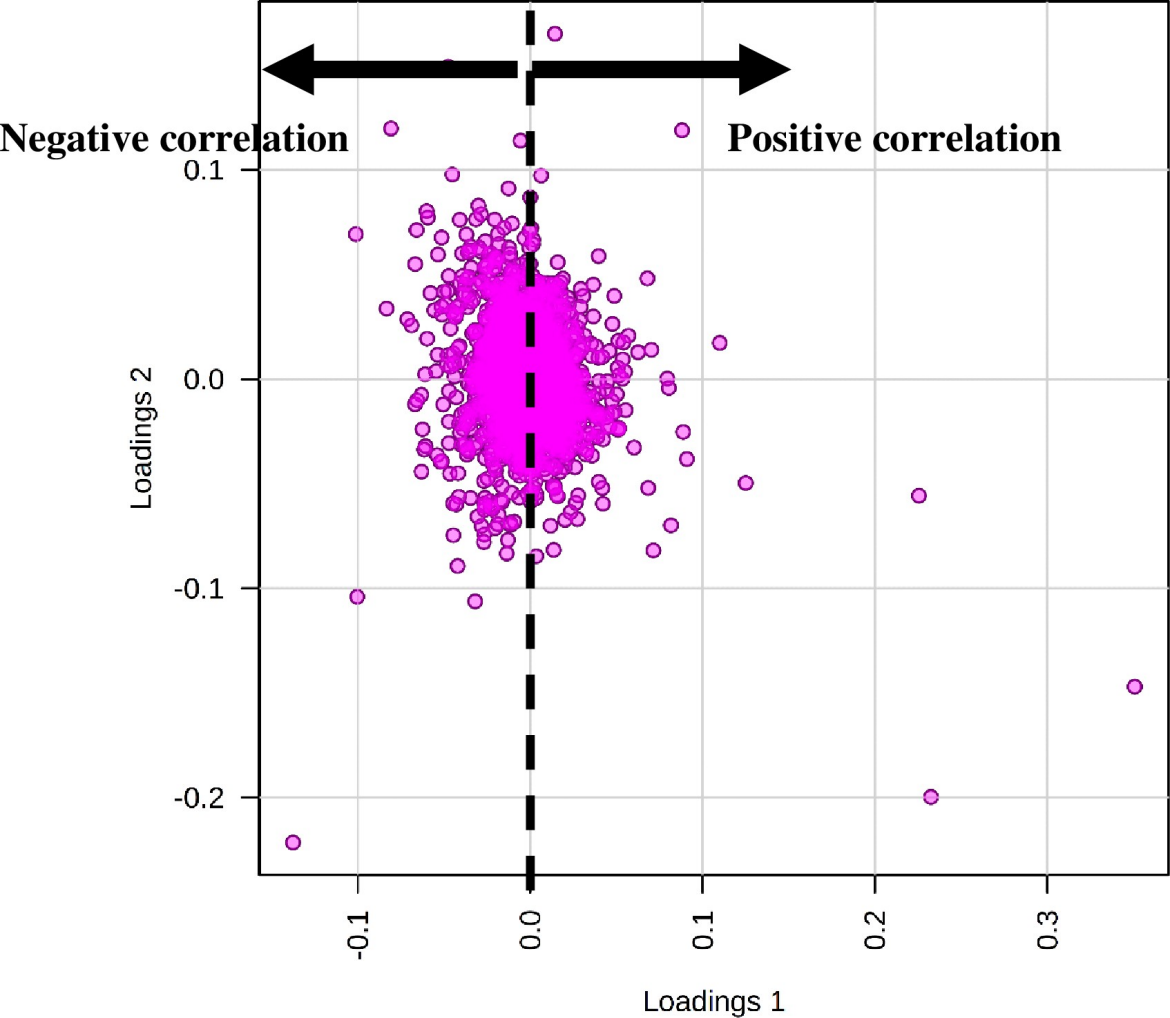
Loadings plot of two principal components from PLS-DA representing proteomics data from the evaluation of four different solubilization buffers. Majority peptides showed no significant difference for each buffer group.

For additional examination of the efficiency variation between all the solubilization buffers, the proteomics data analysis of four replicates corresponded to the four different solubilization buffers were statistically compared using Partial Least Squares Discriminant Analysis (PLS-DA). PLS-DA comprehensively determined the linear relationship between different buffers (*Y* response matrix) and the corresponding peptide spectra (*X* predictor matrix). PLS-DA was applied in the context of our study due to its ability to analyze data with complicated, noisy, collinear and incomplete variables in both *X* and *Y*. The PLS-DA model qualities were cross-validated with a 10-fold cross-validation method based on *R*^*2*^ and *Q*^*2*^ parameters [65]. *R*^*2*^ = 1 is an indication of a perfect data description by the model. In this study, the corresponding *R*^*2*^ and *Q*^*2*^ values for each component were listed in Tables 1 and 2. The value of *R*^*2*^ *–Q*^*2*^ is less than 0.3 for up two components, which indicated that the model has good predictability. The cross-validation correlation coefficient R2, *Q*^*2*^ revealed a value of 0.68067, which was an indicator of a model with high predictive model. A three-component model was the best classifier (S3A Fig). The performed permutation tests, another PLS-DA model cross-validation method, showed that the group separation was statistically insignificant at *p* = 0.451 (S3B Fig). The original model (indicated with red arrow) was part of the 1000 permutated models. The result showed a good elucidation and buffer type classification information [56]. In the supervised PLS-DA of peptide intensities, a clear grouping based on the buffers evaluated were achieved (Fig 6). The model was built between dependent variables (Principal Component 2), represented the urea/thiourea/CHAPS (A), urea/CHAPS (B), urea/SDC (C) and SDC (D) buffers; and independent variables (Principal Component 1) (peptide spectra). The explained variance for the first and second principal components were 25.0% (PC1) and 16.7% (PC2), respectively. Groups of urea/thiourea/CHAPS, urea/CHAPS and urea/SDC were clustered negatively. However, SDC buffer group was positively clustered compared to the rest of the buffer groups. Clearer correlation between the buffer groups was projected in a three-dimensional scores plot, based on three principal components (PC1, 2 and 3) (Fig 7). In this model, it was apparent that urea/thiourea/CHAPS, urea/CHAPS buffers and urea/SDC (A, B and C) were closely related to each other, suggesting that the buffers shared similar characteristics. In this study, the characteristic was possibly the urea additive. Conversely, SDC buffer was located away because there was no similarity in the buffer components. Loadings of the buffer groups (A-D) in this study were explained by the first two principal components (PC1 and PC2) (Fig 8). The principal component loadings used to detect variability, showed that the solubilization buffers were indistinguishable by the profile of peptide spectra. Most loadings were clustered together except for several ‘outliers’. The directions of the loading variables indicated that they were positively (to the right of the *x*-axis) and negatively (to the left of the *x*-axis) correlated.

**Table 1:** PLS-DA cross-validation results based on *Q*^*2*^ parameters.

Measure	1 components	2 components	3 components	4 components
Accuracy	0.25	0.25	0.875	0.8125
R^2^	0.80298	0.91373	0.98337	0.9983
**Q**^**2**^	0.61822	0.67962	0.68067	0.67425

**Table 2:** PLS-DA cross-validation results based on *R*^*2*^ parameters.

Measure	1 components	2 components	3 components	4 components
Accuracy	0.25	0.25	0.875	0.8125
**R**^**2**^	0.80298	0.91373	0.98337	0.99883
Q^2^	0.51011	0.65335	0.62915	0.62121

All the four buffers were able to solubilize the extracted proteins from oil palm fruit mesocarps to a variety of extents. A minimum concentration of 4% (w/v) SDC was used in this study as any concentration less than 4% would not able to solubilize the proteins completely (based on qualitative observations, data not shown). The fluctuation in the total protein yields and electrophoretic profile indicated that urea/thiourea/CHAPS buffer remained the most effective solubilization buffer. Nonetheless, the total protein yields determined from all the solubilization buffers were still satisfactory and the difference was only 0.2 μg/μL between urea/sodium deoxycholate and sodium deoxycholate buffers and urea/thiourea/CHAPS and urea /CHAPS buffers. For urea/CHAPS and urea/SDC comparisons to determine additive effects on protein solubilization, CHAPS–containing buffer was more proficient to solubilize the proteins. However, when chromatogram, spectra, identified protein and peptide numbers, gene ontologies of all the solubilization buffers were compared, at 4% (w/v), SDC alone was broadly applicable to the oil palm mesocarp proteins, despite the lower protein yield and additive efficiency. Detailed statistical approach to analyze oil palm proteomics datasets was also presented in this study. The results were in agreement with the mass spectrometric analysis that there were only minor variations (based on the group clustering) between the different solubilization buffers. These results were significant as four replicates (n = 4) were used for each buffer in the PLS-DA. Inability to find similar experimental set-up in the literatures prevented the comparison of the results acquired from this study in accessing the solubilization efficiency. Currently, SDC has only been used to solubilize membrane proteins [40–43] and to enhance tryptic protein digestion [44–48]. SDC is an acid-removable detergent that able to disrupt cell membranes and protein to protein interactions, similar to sodium dodecyl sulphate. The major advantage of employing SDC is that the detergent is removable through acid precipitation either before or after enzymatic digestion without causing any loss or variability to protein identification rate [46, 66]. Removal of sodium dodecyl sulphate, urea, CHAPS using filter-aided sample preparation (FASP) and zip tips [66–68] still resulted in interferences to liquid chromatography runs and mass spectrometric analysis [68, 69]. The limitation of the utilization of SDC in protein solubilization, which was observed from the study, was the interference to the protein quantitation. However, this limitation could be circumvented by incorporating a 4-fold dilution prior to protein content determination using a colorimetric approach. Alternatively, the proteins could be precipitated before quantitation. Finally, further studies are necessary to determine if SDC could also be applied to animal and human-based proteins for solubilization.

## Conclusions

The study presented a first-time assessment of the utilization of SDC in solubilizing oil palm mesocarp proteins for proteomics studies. While the use of SDC buffer resulted in slightly lower protein yield compared to urea/thiourea/CHAPS buffer, the electrophoretic pattern did not alter extensively. The study had also demonstrated that, in the presence of urea, the efficiency of SDC had only varied slightly compared to CHAPS in assisting protein solubility. However, the mass spectrometric and statistical analyses revealed that SDC, as a buffer, was applicable in oil palm mesocarp protein solubilization and had better efficiency compared to urea/thiourea/CHAPS buffer. Most importantly, unlike the combination of urea, thiourea and CHAPS, SDC has been experimentally proven to increase the number of identified proteins by 135 proteins (421 peptides), enhanced the spectra qualities and major gene ontologies. Remarkably, the occurrence of carbamylation modification on lysine residues of the identified peptides, which could lead to false protein identifications, was reduced by more than 67.0% when SDC buffer was used in protein solubilization. SDC does not need to be removed prior to any mass spectrometric analysis (in contrast with other urea/CHAPS-containing buffers). This advantage alone was able to simplify the sample processing process and improve protein recovery. These cumulative advantages rendered the inexpensive SDC a more effective and economical solubilization buffer in studying the oil palm mesocarp proteins using high-throughput proteomics approach, and not limited to the applications in membrane proteins and enhancement of protein digestion. It was also worth investigating the applicability of SDC in human and animal-extracted proteins for general solubilization.

## Supporting information

S1 Fig Total yields (mean ± SD) of proteins solubilized in four different solubilization buffers (A-D). Buffer A: Urea/thiourea/CHAPS; Buffer B: Urea/CHAPS; Buffer C: Urea/sodium deoxycholate; Buffer D: Sodium deoxycholate.
(TIF)

S2 Fig One-dimensional gel electrophoresis (SDS-PAGE) of the proteins solubilized in four different solubilization buffers (A-D). 100 μg was loaded for protein separation and the SDS-PAGE gel was stained with an in-house prepared colloidal Coomassie G-250. M and numbers on the gel represent the Merck Perfect Protein Markers (protein ladders) used and kiloDalton, respectively. Red box indicates loss of protein bands for Buffer D. Buffer A: Urea/thiourea/CHAPS; Buffer B: Urea/CHAPS; Buffer C: Urea/Sodium deoxycholate; Buffer D: Sodium deoxycholate.
(TIF)

S3 Fig (A) PLS-DA cross-validation result and (B) Permutation test statistics. The selected Q2 performance measure indicated that a four-component model is the best classifier (red star). The permutation tests consisted of 1000 permutations and showed that the group separation was statistically significant at p = 0.451 (red arrow).
(TIF)

S1 File Modification profile of peptides identified. (XLSX)

S2 File Uniprot ID used for gene ontology annotation. (XLSX)

## References

[1] Kushairi A, Loh SK, Azman I, Hishamuddin E, Ong Abdullah M, Mohd Noor Izuddin ZB, et al Oil palm economic performance in Malaysia and R&D progress in 2017. Journal of Oil Palm Research. 2018;30(2):163–95. 10.21894/jopr.2018.0030

[2] Loei H, Lim J, Tan M, Lim TK, Lin QS, Chew FT, et al Proteomic analysis of the oil palm fruit mesocarp reveals elevated oxidative phosphorylation activity is critical for increased storage oil production. J Proteome Res. 2013;12(11):5096–109. Epub 2013/10/03. 10.1021/pr400606h . 24083564

[3] Lau BY, Clerens S, Morton JD, Dyer JM, Deb-Choudhury S, Ramli US. Application of a Mass Spectrometric Approach to Detect the Presence of Fatty Acid Biosynthetic Phosphopeptides. Protein J. 2016;35(2):163–70. Epub 2016/03/20. 10.1007/s10930-016-9655-0 . 26993480

[4] Lau BYC, Morton DJ, Deb-Choudhury S, Clerens S, Dyer JM, Ramli US. Differential expression analysis of oil palm fatty acid biosynthetic enzymes with gel-free quantitative proteomics. Journal of Oil Palm Research. 2017;29(1):23–34.

[5] Ooi TE, Yeap WC, Daim LD, Ng BZ, Lee FC, Othman AM, et al Differential abundance analysis of mesocarp protein from high- and low-yielding oil palms associates non-oil biosynthetic enzymes to lipid biosynthesis. Proteome Sci. 2015;13(1):28 Epub 2015/12/01. 10.1186/s12953-015-0085-2 26617468 PMC4661986

[6] Tan HS, Liddell S, Ong Abdullah M, Wong WC, Chin CF. Differential proteomic analysis of embryogenic lines in oil palm (*Elaeis guineensis* Jacq). J Proteomics. 2016;143:334–45. 10.1016/j.jprot.2016.04.039 . 27130535

[7] Al-Obaidi JR, Mohd-Yusuf Y, Razali N, Jayapalan JJ, Tey CC, Md-Noh N, et al Identification of proteins of altered abundance in oil palm infected with *Ganoderma boninense*. Int J Mol Sci. 2014;15(3):5175–92. 10.3390/ijms15035175 24663087 PMC3975447

[8] Daim LDJ, Ooi TE, Ithnin N, Mohd Yusof H, Kulaveerasingam H, Abdul Majid N, et al Comparative proteomic analysis of oil palm leaves infected with *Ganoderma boninense* revealed changes in proteins involved in photosynthesis, carbohydrate metabolism, and immunity and defense. Electrophoresis. 2015;36(15):1699–710. 10.1002/elps.201400608 . 25930948

[9] Adam H, Jouannic S, Morcillo F, Verdeil JL, Duval Y, Tregear JW. Determination of flower structure in *Elaeis guineensis*: do palms use the same homeotic genes as other species? Ann Bot. 2007;100(1):1–12. Epub 2007/03/16. 10.1093/aob/mcm027 17355996 PMC2735288

[10] Singh R, Low ET, Ooi LC, Ong-Abdullah M, Ting NC, Nagappan J, et al The oil palm SHELL gene controls oil yield and encodes a homologue of SEEDSTICK. Nature. 2013;500(7462):340–4. 10.1038/nature12356 23883930 PMC4209285

[11] Singh R, Ong-Abdullah M, Low ET, Manaf MA, Rosli R, Nookiah R, et al Oil palm genome sequence reveals divergence of interfertile species in Old and New worlds. Nature. 2013;500(7462):335–9. 10.1038/nature12309 23883927 PMC3929164

[12] Dussert S, Guerin C, Andersson M, Joet T, Tranbarger TJ, Pizot M, et al Comparative transcriptome analysis of three oil palm fruit and seed tissues that differ in oil content and fatty acid composition. Plant Physiol. 2013;162(3):1337–58. Epub 2013/06/06. 10.1104/pp.113.220525 23735505 PMC3707537

[13] Tranbarger TJ, Dussert S, Joet T, Argout X, Summo M, Champion A, et al Regulatory mechanisms underlying oil palm fruit mesocarp maturation, ripening, and functional specialization in lipid and carotenoid metabolism. Plant Physiol. 2011;156(2):564–84. Epub 2011/04/14. 10.1104/pp.111.175141 21487046 PMC3177259

[14] Bourgis F, Kilaru A, Cao X, Ngando-Ebongue GF, Drira N, Ohlrogge JB, et al Comparative transcriptome and metabolite analysis of oil palm and date palm mesocarp that differ dramatically in carbon partitioning. Proc Natl Acad Sci U S A. 2011;108(30):12527–32. Epub 2011/06/29. 10.1073/pnas.1106502108 21709233 PMC3145713

[15] Morcillo F, Cros D, Billotte N, Ngando-Ebongue GF, Domonhedo H, Pizot M, et al Improving palm oil quality through identification and mapping of the lipase gene causing oil deterioration. Nat Commun. 2013;4:2160 Epub 2013/07/17. 10.1038/ncomms3160 23857501 PMC3717496

[16] Lau BYC. Proteomic profiling of fatty acid biosynthetic enzymes from oil palm chromoplast. Lincoln, New Zealand: Lincoln University; 2015.

[17] Ye X, Li L. Microwave-assisted protein solubilization for mass spectrometry-based shotgun proteome analysis. Analytical Chemistry. 2012;84(14):6181–91. 10.1021/ac301169q 22708679

[18] Wang W, Scali M, Vignani R, Spadafora A, Sensi E, Mazzuca S, et al Protein extraction for two-dimensional electrophoresis from olive leaf, a plant tissue containing high levels of interfering compounds. ELECTROPHORESIS. 2003;24(14):2369–75. 10.1002/elps.200305500 12874872

[19] Wang W, Vignani R, Scali M, Cresti M. A universal and rapid protocol for protein extraction from recalcitrant plant tissues for proteomic analysis. ELECTROPHORESIS. 2006;27(13):2782–6. 10.1002/elps.200500722 16732618

[20] Wang W, Vignani R, Scali M, Sensi E, Tiberi P, Cresti M. Removal of lipid contaminants by organic solvents from oilseed protein extract prior to electrophoresis. Analytical Biochemistry. 2004;329(1):139–41. Epub 2004/05/12. 10.1016/j.ab.2004.02.044 . 15136176

[21] Xie H, Pan S, Liu S, Ye K, Huo K. A novel method of protein extraction from perennial *Bupleurum* root for 2-DE. ELECTROPHORESIS. 2007;28(5):871–5. Epub 2007/02/23. 10.1002/elps.200600354 . 17315152

[22] Fan P, Wang X, Kuang T, Li Y. An efficient method for the extraction of chloroplast proteins compatible for 2-DE and MS analysis. ELECTROPHORESIS. 2009;30(17):3024–33. Epub 2009/08/14. 10.1002/elps.200900172 . 19676087

[23] Gomez A, Lopez JA, Pintos B, Camafeita E, Bueno MA. Proteomic analysis from haploid and diploid embryos of *Quercus suber* L. identifies qualitative and quantitative differential expression patterns. Proteomics. 2009;9(18):4355–67. Epub 2009/08/08. 10.1002/pmic.200900179 . 19662628

[24] He C-F, Wang Y-M. Protein extraction from leaves of *Aloe vera* L., a succulent and recalcitrant plant, for proteomic analysis. Plant Molecular Biology Reporter. 2008;26(4):292–300. 10.1007/s11105-008-0040-9

[25] Hao R, Adoligbe C, Jiang B, Zhao X, Gui L, Qu K, et al An optimized trichloroacetic acid/acetone precipitation method for two-dimensional gel electrophoresis analysis of Qinchuan cattle longissimus dorsi muscle containing high proportion of marbling. PLoS One. 2015;10(4):e0124723 Epub 2015/04/22. 10.1371/journal.pone.0124723 25893432 PMC4404140

[26] Isaacson T, Damasceno CM, Saravanan RS, He Y, Catala C, Saladie M, et al Sample extraction techniques for enhanced proteomic analysis of plant tissues. Nature protocols. 2006;1(2):769–74. Epub 2007/04/05. 10.1038/nprot.2006.102 . 17406306

[27] Santos CA, Beloti LL, Toledo MA, Crucello A, Favaro MT, Mendes JS, et al A novel protein refolding protocol for the solubilization and purification of recombinant peptidoglycan-associated lipoprotein from *Xylella fastidiosa* overexpressed in *Escherichia coli*. Protein Expr Purif. 2012;82(2):284–9. Epub 2012/02/07. 10.1016/j.pep.2012.01.010 . 22306742

[28] Yang Z, Zhang L, Zhang Y, Zhang T, Feng Y, Lu X, et al Highly efficient production of soluble proteins from insoluble inclusion bodies by a two-step-denaturing and refolding method. PLoS One. 2011;6(7):e22981 Epub 2011/08/11. 10.1371/journal.pone.0022981 21829569 PMC3146519

[29] Bennion BJ, Daggett V. The molecular basis for the chemical denaturation of proteins by urea. Proceedings of the National Academy of Sciences. 2003;100(9):5142–7. 10.1073/pnas.0930122100 12702764 PMC154312

[30] Gang D, Xinyue Z, Na Z, Chengying L, Bo W, Dingxiang P, et al A proteomics sample preparation method for mature, recalcitrant leaves of perennial plants. PLoS One. 2014;9(7):e102175 Epub 2014/07/17. 10.1371/journal.pone.0102175 25028960 PMC4100801

[31] Wu Y, Zhou J, Zhang X, Zheng X, Jiang X, Shi L, et al Optimized sample preparation for two-dimensional gel electrophoresis of soluble proteins from chicken bursa of Fabricius. Proteome Science. 2009;7(1):38 Epub 2009/10/10. 10.1186/1477-5956-7-38 19814785 PMC2770044

[32] Yu YQ, Gilar M, Lee PJ, Bouvier ES, Gebler JC. Enzyme-friendly, mass spectrometry-compatible surfactant for in-solution enzymatic digestion of proteins. Anal Chem. 2003;75(21):6023–8. Epub 2003/11/01. 10.1021/ac0346196 . 14588046

[33] Lu X, Zhu H. Tube-gel digestion: a novel proteomic approach for high throughput analysis of membrane proteins. Molecular & cellular proteomics: MCP. 2005;4(12):1948–58. Epub 2005/09/10. 10.1074/mcp.M500138-MCP200 16150870 PMC1360194

[34] Gorg A, Weiss W, Dunn MJ. Current two-dimensional electrophoresis technology for proteomics. Proteomics. 2004;4(12):3665–85. Epub 2004/11/16. 10.1002/pmic.200401031 . 15543535

[35] Gundry R L., White M Y., Murray C I., Kane L A., Fu Q, Stanley B, A., et al Preparation of proteins and peptides for mass spectrometry analysis in a bottom-up proteomics workflow. Current protocols in Molecular Biology. 2009;88:10.25.1–10.25.3. 10.1002/0471142727.mb1025s88 19816929 PMC2905857

[36] Scheerlinck E, Dhaenens M, Van Soom A, Peelman L, De Sutter P, Van Steendam K, et al Minimizing technical variation during sample preparation prior to label-free quantitative mass spectrometry. Analytical Biochemistry. 2015;490:14–9. Epub 2015/08/25. 10.1016/j.ab.2015.08.018 . 26302362

[37] Kollipara L, Zahedi RP. Protein carbamylation: in vivo modification or in vitro artefact? Proteomics. 2013;13(6):941–4. Epub 2013/01/22. 10.1002/pmic.201200452 . 23335428

[38] Chen EI, Cociorva D, Norris JL, Yates JR. Optimization of mass spectrometry-compatible surfactants for shotgun proteomics. Journal of Proteome Research. 2007;6(7):2529–38. 10.1021/pr060682a 17530876 PMC2570269

[39] Poulsen JW, Madsen CT, Young C, Poulsen FM, Nielsen ML. Using guanidine-hydrochloride for fast and efficient protein digestion and single-step affinity-purification mass spectrometry. J Proteome Res. 2013;12(2):1020–30. Epub 2012/11/29. 10.1021/pr300883y . 23186134

[40] Zhou J, Zhou T, Cao R, Liu Z, Shen J, Chen P, et al Evaluation of the application of sodium deoxycholate to proteomic analysis of rat hippocampal plasma membrane. Journal of Proteome Research. 2006;5(10):2547–53. Epub 2006/10/07. 10.1021/pr060112a . 17022626

[41] Lin Y, Liu Y, Li J, Zhao Y, He Q, Han W, et al Evaluation and optimization of removal of an acid-insoluble surfactant for shotgun analysis of membrane proteome. ELECTROPHORESIS. 2010;31(16):2705–13. 10.1002/elps.201000161 20665523

[42] Odahara T. Stability and solubility of integral membrane proteins from photosynthetic bacteria solubilized in different detergents. Biochimica et Biophysica Acta (BBA)—Biomembranes. 2004;1660(1–2):80–92. 10.1016/j.bbamem.2003.11.003 14757223

[43] Masuda T, Tomita M, Ishihama Y. Phase transfer surfactant-aided trypsin digestion for membrane proteome analysis. Journal of Proteome Research. 2008;7(2):731–40. Epub 2008/01/11. 10.1021/pr700658q . 18183947

[44] Koehn H, Lau B, Clerens S, Plowman JE, Dyer JM, Ramli US, et al Combination of acid labile detergent and C18 Empore disks for improved identification and sequence coverage of in-gel digested proteins. Anal Bioanal Chem. 2011;400(2):415–21. Epub 2011/02/18. 10.1007/s00216-011-4765-1 . 21327873

[45] Lin Y, Wang K, Liu Z, Lin H, Yu L. Enhanced SDC-assisted digestion coupled with lipid chromatography-tandem mass spectrometry for shotgun analysis of membrane proteome. Journal of chromatography B, Analytical technologies in the biomedical and life sciences. 2015;1002:144–51. Epub 2015/09/01. 10.1016/j.jchromb.2015.08.019 26319803

[46] Lin Y, Zhou J, Bi D, Chen P, Wang X, Liang S. Sodium-deoxycholate-assisted tryptic digestion and identification of proteolytically resistant proteins. Analytical Biochemistry. 2008;377(2):259–66. 10.1016/j.ab.2008.03.009 18384734

[47] Proc JL, Kuzyk MA, Hardie DB, Yang J, Smith DS, Jackson AM, et al A quantitative study of the effects of chaotropic agents, surfactants, and solvents on the digestion efficiency of human plasma proteins by trypsin. Journal of Proteome Research. 2010;9(10):5422–37. 10.1021/pr100656u 20722421 PMC2996461

[48] Glatter T, Ahrne E, Schmidt A. Comparison of Different Sample Preparation Protocols Reveals Lysis Buffer-Specific Extraction Biases in Gram-Negative Bacteria and Human Cells. Journal of Proteome Research. 2015;14(11):4472–85. Epub 2015/09/29. 10.1021/acs.jproteome.5b00654 . 26412744

[49] Lau BY, Deb-Choudhury S, Morton JD, Clerens S, Dyer JM, Ramli US. Method developments to extract proteins from oil palm chromoplast for proteomic analysis. Springerplus. 2015;4(1):791 Epub 2015/12/25. 10.1186/s40064-015-1576-4 26702380 PMC4688294

[50] Perez-Riverol Y, Csordas A, Bai J, Bernal-Llinares M, Hewapathirana S, Kundu DJ, et al The PRIDE database and related tools and resources in 2019: improving support for quantification data. Nucleic Acids Res. 2019;47(D1):D442–D50. Epub 2018/11/06. 10.1093/nar/gky1106 30395289 PMC6323896

[51] Xia J, Sinelnikov IV, Han B, Wishart DS. MetaboAnalyst 3.0—making metabolomics more meaningful. Nucleic Acids Research. 2015;43(Web Server issue):W251–W7. 10.1093/nar/gkv380 PMC4489235. 25897128 PMC4489235

[52] Hackstadt AJ, Hess AM. Filtering for increased power for microarray data analysis. BMC Bioinformatics. 2009;10(1):11–23. Epub 2009/01/10. 10.1186/1471-2105-10-11 19133141 PMC2661050

[53] van den Berg RA, Hoefsloot HC, Westerhuis JA, Smilde AK, van der Werf MJ. Centering, scaling, and transformations: improving the biological information content of metabolomics data. BMC Genomics. 2006;7(1):142–53. Epub 2006/06/10. 10.1186/1471-2164-7-142 16762068 PMC1534033

[54] Wheelock AM, Wheelock CE. Trials and tribulations of 'omics data analysis: assessing quality of SIMCA-based multivariate models using examples from pulmonary medicine. Mol Biosyst. 2013;9(11):2589–96. 10.1039/c3mb70194h . 23999822

[55] Worley B, Powers R. Multivariate analysis in metabolomics. Curr Metabolomics. 2013;1(1):92–107. Epub 2013/01/01. 10.2174/2213235X11301010092 26078916 PMC4465187

[56] Bijlsma S, Bobeldijk I, Verheij ER, Ramaker R, Kochhar S, Macdonald IA, et al Large-scale human metabolomics studies: a strategy for data (pre-) processing and validation. Anal Chem. 2006;78(2):567–74. Epub 2006/01/18. 10.1021/ac051495j . 16408941

[57] Barberini L, Noto A, Saba L, Palmas F, Fanos V, Dessì A, et al Multivariate data validation for investigating primary HCMV infection in pregnancy. Data in Brief. 2016;9:220–30. 10.1016/j.dib.2016.08.050 PMC5021794. 27656676 PMC5021794

[58] Malafaia CB, Guerra ML, Silva TD, Paiva PM, Souza EB, Correia MT, et al Selection of a protein solubilization method suitable for phytopathogenic bacteria: a proteomics approach. Proteome Sci. 2015;13:5–17. Epub 2015/02/12. 10.1186/s12953-015-0062-9 25670925 PMC4322814

[59] Dyer JM, Stymne S, Green AG, Carlsson AS. High-value oils from plants. The Plant Journal. 2008;54(4):640–55. 10.1111/j.1365-313X.2008.03430.x 18476869

[60] Lau B, Othman A. Transcriptomics and Proteomics Analysis of Fatty Acid Regulation in Oil Palm. In preparation. 2019.

[61] Broeckx V, Boonen K, Pringels L, Sagaert X, Prenen H, Landuyt B, et al Comparison of multiple protein extraction buffers for GeLC-MS/MS proteomic analysis of liver and colon formalin-fixed, paraffin-embedded tissues. Molecular BioSystems. 2016;12(2):553–65. 10.1039/c5mb00670h 26676081

[62] Urbaneja MA, Alonso A, Gonzalez-Manas JM, Goni FM, Partearroyo MA, Tribout M, et al Detergent solubilization of phospholipid vesicle. Effect of electric charge. Biochem J. 1990;270(2):305–8. Epub 1990/09/01. 10.1042/bj2700305 2400390 PMC1131720

[63] Churchward MA, Butt RH, Lang JC, Hsu KK, Coorssen JR. Enhanced detergent extraction for analysis of membrane proteomes by two-dimensional gel electrophoresis. Proteome Science. 2005;3:5–. 10.1186/1477-5956-3-5 PMC1184097. 15941475 PMC1184097

[64] Rodi PM, Bocco Gianello MD, Corregido MC, Gennaro AM. Comparative study of the interaction of CHAPS and Triton X-100 with the erythrocyte membrane. Biochimica et biophysica acta. 2014;1838(3):859–66. Epub 2013/11/19. 10.1016/j.bbamem.2013.11.006 . 24239862

[65] Triba MN, Le Moyec L, Amathieu R, Goossens C, Bouchemal N, Nahon P, et al PLS/OPLS models in metabolomics: the impact of permutation of dataset rows on the K-fold cross-validation quality parameters. Mol Biosyst. 2015;11(1):13–9. 10.1039/c4mb00414k . 25382277

[66] Zhang N, Li L. Effects of common surfactants on protein digestion and matrix-assisted laser desorption/ionization mass spectrometric analysis of the digested peptides using two-layer sample preparation. Rapid Communications in Mass Spectrometry. 2004;18(8):889–96. Epub 2004/04/20. 10.1002/rcm.1423 . 15095358

[67] Wisniewski JR, Zougman A, Nagaraj N, Mann M. Universal sample preparation method for proteome analysis. Nature Methods. 2009;6(5):359–62. Epub 2009/04/21. 10.1038/nmeth.1322 . 19377485

[68] Shevchenko G, Musunuri S, Wetterhall M, Bergquist J. Comparison of extraction methods for the comprehensive analysis of mouse brain proteome using shotgun-based mass spectrometry. Journal of Proteome Research. 2012;11(4):2441–51. Epub 2012/02/23. 10.1021/pr201169q . 22352882

[69] Hustoft HK, Reubsaet L, Greibrokk T, Lundanes E, Malerod H. Critical assessment of accelerating trypsination methods. Journal of Pharmaceutical and Biomedical Analysis. 2011;56(5):1069–78. Epub 2011/08/30. 10.1016/j.jpba.2011.08.013 . 21873015

