# Supplementary figures and images for: Evaluation of sodium deoxycholate as solubilization buffer for oil palm proteomics analysis
Source: PLoS One. 2019 Aug 15;14(8):e0221052. doi: 10.1371/journal.pone.0221052 (PMC6695131; doi:10.1371/journal.pone.0221052)

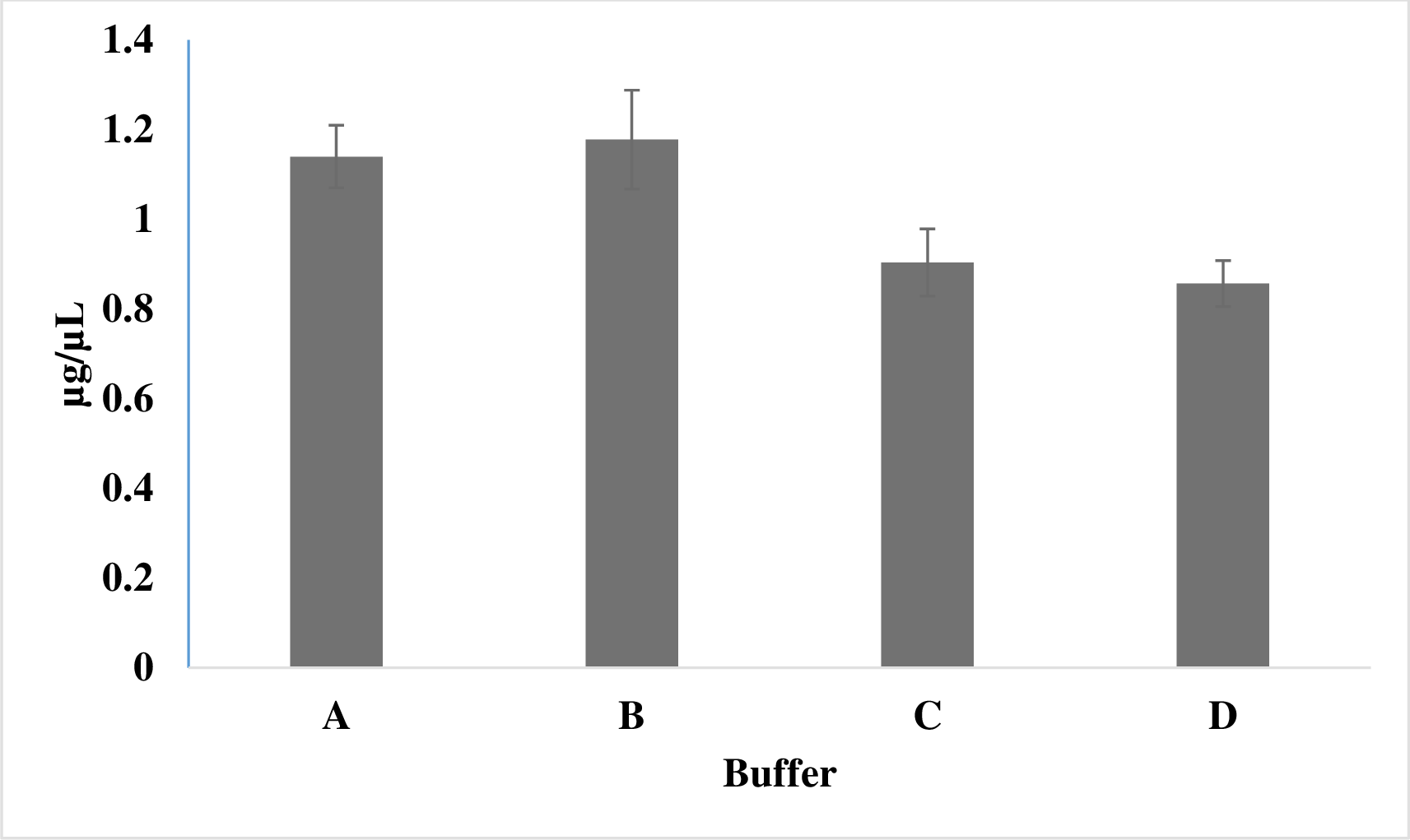

Supplement: S1 Fig — Total yields (mean ± SD) of proteins solubilized in four different solubilization buffers (A-D). Buffer A: Urea/thiourea/CHAPS; Buffer B: Urea/CHAPS; Buffer C: Urea/sodium deoxycholate; Buffer D: Sodium deoxycholate. (TIF) [file pone.0221052.s001.tif]

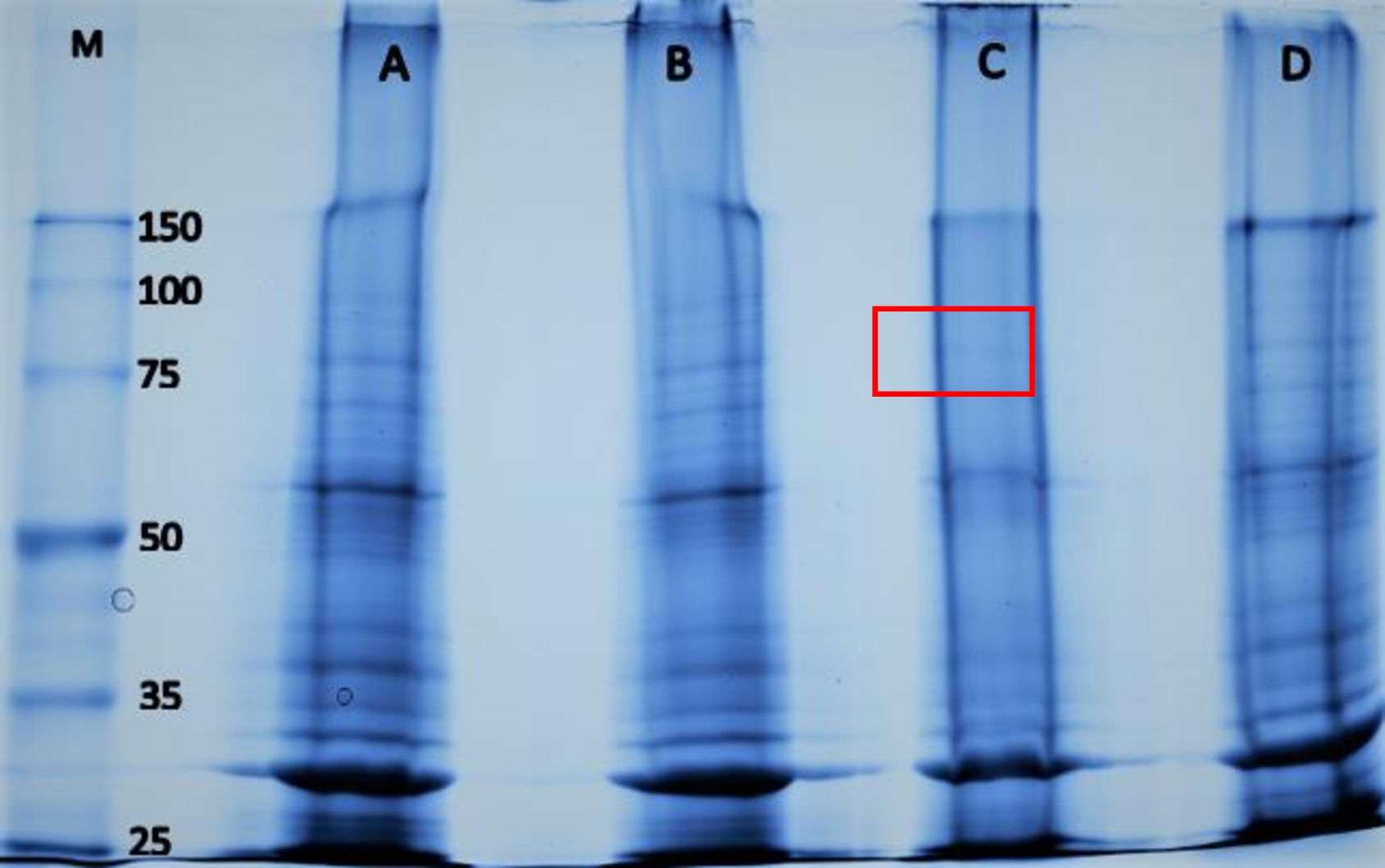

Supplement: S2 Fig — One-dimensional gel electrophoresis (SDS-PAGE) of the proteins solubilized in four different solubilization buffers (A-D). 100 μg was loaded for protein separation and the SDS-PAGE gel was stained with an in-house prepared colloidal Coomassie G-250. M and numbers on the gel represent the Merck Perfect Protein Markers (protein ladders) used and kiloDalton, respectively. Red box indicates loss of protein bands for Buffer D. Buffer A: Urea/thiourea/CHAPS; Buffer B: Urea/CHAPS; Buffer C: Urea/Sodium deoxycholate; Buffer D: Sodium deoxycholate. (TIF) [file pone.0221052.s002.tif]

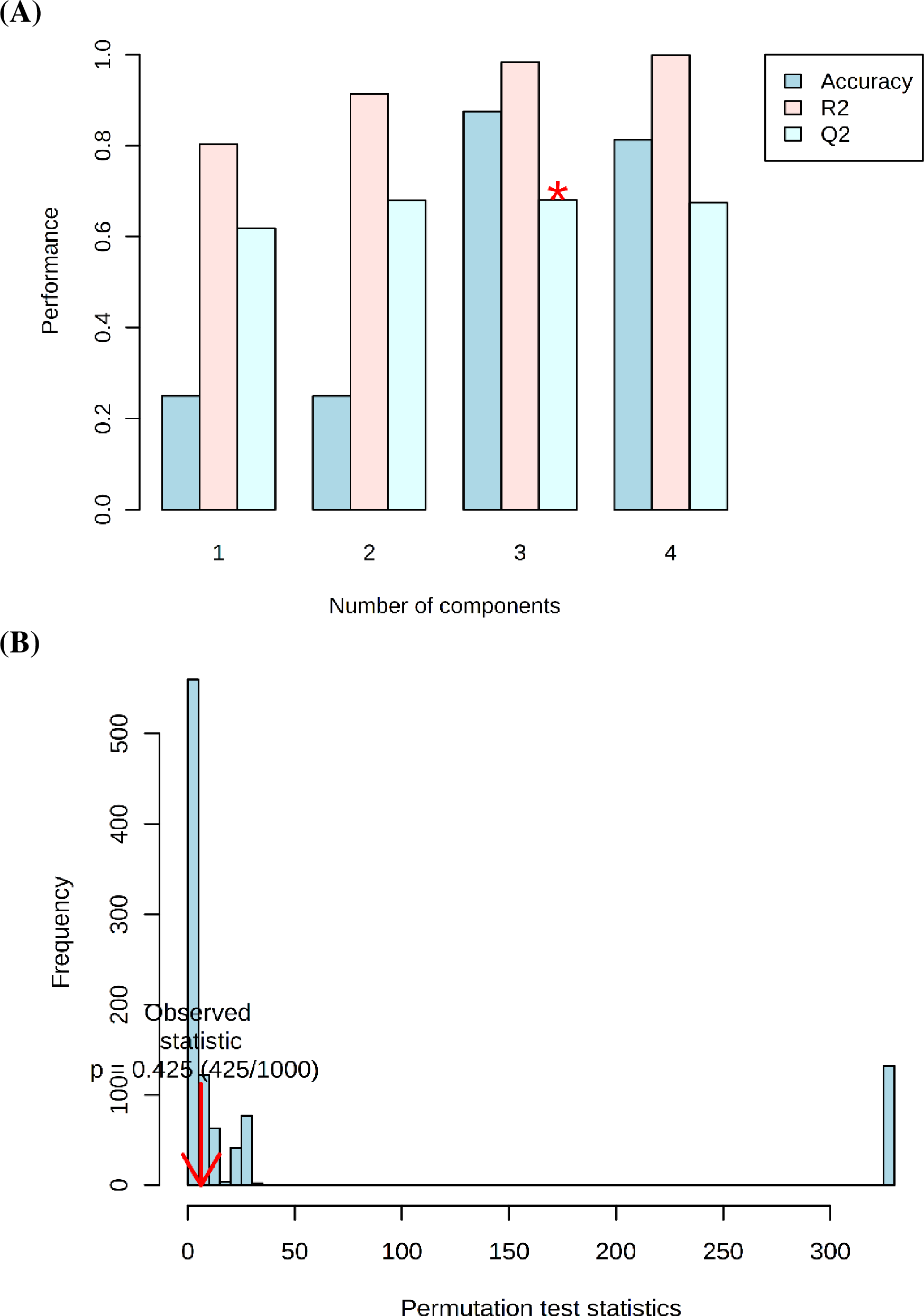

Supplement: S3 Fig — (A) PLS-DA cross-validation result and (B) Permutation test statistics. The selected Q2 performance measure indicated that a four-component model is the best classifier (red star). The permutation tests consisted of 1000 permutations and showed that the group separation was statistically significant at p = 0.451 (red arrow). (TIF) [file pone.0221052.s003.tif]
